# Diagnostic value of transpulmonary thermodilution measurements for acute respiratory distress syndrome in a pig model of septic shock

**DOI:** 10.1186/s12967-022-03793-x

**Published:** 2022-12-23

**Authors:** Yusuke Endo, Taku Miyasho, Kanako Endo, Yoshio Kawamura, Kenjiro Miyoshi, Ryosuke Takegawa, Takashi Tagami, Lance B. Becker, Kei Hayashida

**Affiliations:** 1 The Feinstein Institutes for Medical Research, Northwell Health System, 350 Community Drive, Manhasset, NY 11030 USA; 2 School of Veterinary Medicine, Rakuno Gakuen University, Ebetsu, Hokkaido Japan; 3 Department of Emergency Medicine and Critical Care Medicine, Nippon Medical School Musashikosugi Hospital, Kawasaki, Kanagawa Japan; 4 Department of Emergency Medicine, Donald and Barbara Zucker School of Medicine at Hofstra/Northwell, Hempstead, NY USA; 5 Department of Emergency Medicine, South Shore University Hospital, Northwell Health, Bay Shore, NY USA

**Keywords:** Pulmonary vascular permeability index, ARDS, Extravascular lung water, Diagnostic criteria, Large animal study

## Abstract

**Background:**

No direct approach assessing pulmonary vascular permeability exists in the current therapeutic strategy for patients with acute respiratory distress syndrome (ARDS). Transpulmonary thermodilution measures hemodynamic parameters such as pulmonary vascular permeability index and extravascular lung water, enabling clinicians to assess ARDS severity. The aim of this study is to explore a precise transpulmonary thermodilution-based criteria for quantifying the severity of lung injury using a clinically relevant septic-ARDS pig model.

**Methods:**

Thirteen female pigs (weight: 31 ± 2 kg) were intubated, mechanically ventilated under anesthesia, and either assigned to septic shock-induced ARDS or control group. To confirm the development of ARDS, we performed computed tomography (CT) imaging in randomly selected animals. The pulmonary vascular permeability index, extravascular lung water, and other hemodynamic parameters were consecutively measured during the development of septic lung injury. Lung status was categorized as normal (partial pressure of oxygen/fraction of inspired oxygen ≥ 400), or injured at different degrees: pre-ARDS (300–400), mild-to-moderate ARDS (100–300), or severe ARDS (< 100). We also measured serum inflammatory cytokines and high mobility group box 1 levels during the experiment to explore the relationship of the pulmonary vascular permeability index with these inflammatory markers.

**Results:**

Using CT image, we verified that animals subjected to ARDS presented an extent of consolidation in bilateral gravitationally dependent gradient that expands over time, with diffuse ground-glass opacification. Further, the post-mortem histopathological analysis for lung tissue identified the key features of diffuse alveolar damage in all animals subjected to ARDS. Both pulmonary vascular permeability index and extravascular lung water increased significantly, according to disease severity. Receiver operating characteristic analysis demonstrated that a cut-off value of 3.9 for the permeability index provided optimal sensitivity and specificity for predicting severe ARDS (area under the curve: 0.99, 95% confidence interval, 0.98–1.00; sensitivity = 100%, and specificity = 92.5%). The pulmonary vascular permeability index was superior in its diagnostic value than extravascular lung water. Furthermore, the pulmonary vascular permeability index was significantly associated with multiple parameters reflecting clinicopathological changes in animals with ARDS.

**Conclusion:**

The pulmonary vascular permeability index is an effective indicator to measure septic ARDS severity.

**Supplementary Information:**

The online version contains supplementary material available at 10.1186/s12967-022-03793-x.

## Introduction

Acute respiratory distress syndrome (ARDS) is a life-threatening condition characterized by non-cardiogenic pulmonary edema and rapid progressive diffuse alveolar damage (DAD) [1]. Despite progress in lung protective strategies, ARDS is associated with high mortality, which ranges from 35–46% among patients [2]. There is no effective pharmacotherapy to supplement lung protective ventilation management for improving patient outcomes in ARDS. This can be attributed to the absence of a precise definition of ARDS [3, 4]. Unfortunately, the current “Berlin definition” is not useful for assessing lung severity [5, 6], thereby increasing the difficulties in the management of patients with ARDS. Of note, among all patients who met the Berlin definition of ARDS, only 45% had DAD [7]. Therefore, the development of direct and highly reproducible approaches for assessing pulmonary vascular permeability and extravascular lung water (EVLW) is an unmet medical need.

Optimal fluid management is crucial in critically ill patients [8–10]. Transpulmonary thermodilution (TPTD) is an established technique for comprehensively measuring the hemodynamic parameters, extravascular lung volume, and lung permeability at the bedside [8, 11–13]. A body of evidence have shown the clinical usefulness of the TPTD technique for quantitative measurement of EVLW and pulmonary vascular permeability in previous decades [14–20]. The normal range of the EVLW indexed to the actual body weight (i.e., extravascular lung water index: ELWI) is < 10 mL/kg, according to early human observational studies [14, 15]. Higher pulmonary vascular permeability is another hallmark of ARDS and can be assessed by calculating the pulmonary vascular permeability index (PVPI), which refers to the ratio between the ELWI and pulmonary blood volume [11, 14, 18, 21]. PVPI obtained using a TPTD is the only procedure to estimate the degree of lung permeability at the bedside. Currently, a PVPI value > 3 (concurrent with ELWI > 10 mL/kg) is widely used in clinical settings as the threshold suggestive of permeability pulmonary edema or ARDS [12]. This threshold has been based on early human observational studies [14, 17, 18]. However, the PVPI is calculated from the ELWI and pulmonary blood volume. Distributive shock and/or severe cardiac dysfunction would affect the threshold in terms of ARDS diagnosis during septic shock. Nevertheless, no animal or human studies has investigated the PVPI threshold for detecting ARDS during septic shock.

Herein, we intended to demonstrate a highly reproducible pig model of sepsis-induced ARDS with hypodynamic shock, induced by “two hits” of lipopolysaccharide (LPS) injections under mechanical ventilation with 100% oxygen. We aimed to explore a precise TPTD-based criteria for quantifying the severity of lung injury using this clinically relevant septic-ARDS model. We hypothesized that PVPI can be a feasible and useful indicator to detect the severity of ARDS during septic shock.

## Methods

This study was approved by the ethics committee for animal experiments at the Rakuno Gakuen University (Protocol Number, VH19B14). The care and handling of the animals were performed in accordance with the guidelines of the National Institutes of Health.

### Animal preparation

We used thirteen healthy LWD pigs (aged 3–4 weeks, weight 29–34 kg). Animals were premedicated with an intramuscular injection of medetomidine hydrochloride (40.0 µg/kg), midazolam (0.2 mg/kg), and butorphanol tartrate (0.2 mg/kg). They underwent tracheal intubation following anesthesia using propofol (6 mg/kg). We maintained general anesthesia with 2% sevoflurane (Sevoflo^®^, Dainippon-Sumitomo Pharma, Osaka, Japan) during the experiment. Neuromuscular blockade was achieved by vecuronium (2 mg/kg, Musculate^®^, Fuji Pharma Co., Tokyo, Japan) and maintained by its continuous infusion (0.1 mg/kg/h) throughout the experiment. All animals were mechanically ventilated in the volume-control mode (10 mL/kg, Flow-i, Maquet, Sonia, Sweden). The right femoral artery was catheterized with a thermistor-tipped 4 Fr Pulse index Continuous Cardiac Output (PiCCO) catheter (PV2014L16, Pulsion Medical Systems AG, Munich, Germany) connected to a Pulsioflex (Getinge, Göteborg, Sweden). We inserted a 6 Fr double-lumen central venous catheter (UK catheter kit UB-0610-W, 21G, 10 cm, Unitika Medical, Osaka, Japan) into the right jugular vein, positioned at the cranial end of the superior vena cava for injecting 0.9% of ice-cold saline. The distal ports of double lumen catheter were connected to the PiCCO_2_ sensor for the TPTD. All transducers were zeroed and positioned at the level of the right atrium.

### Experimental protocol

**Fig. 1:**
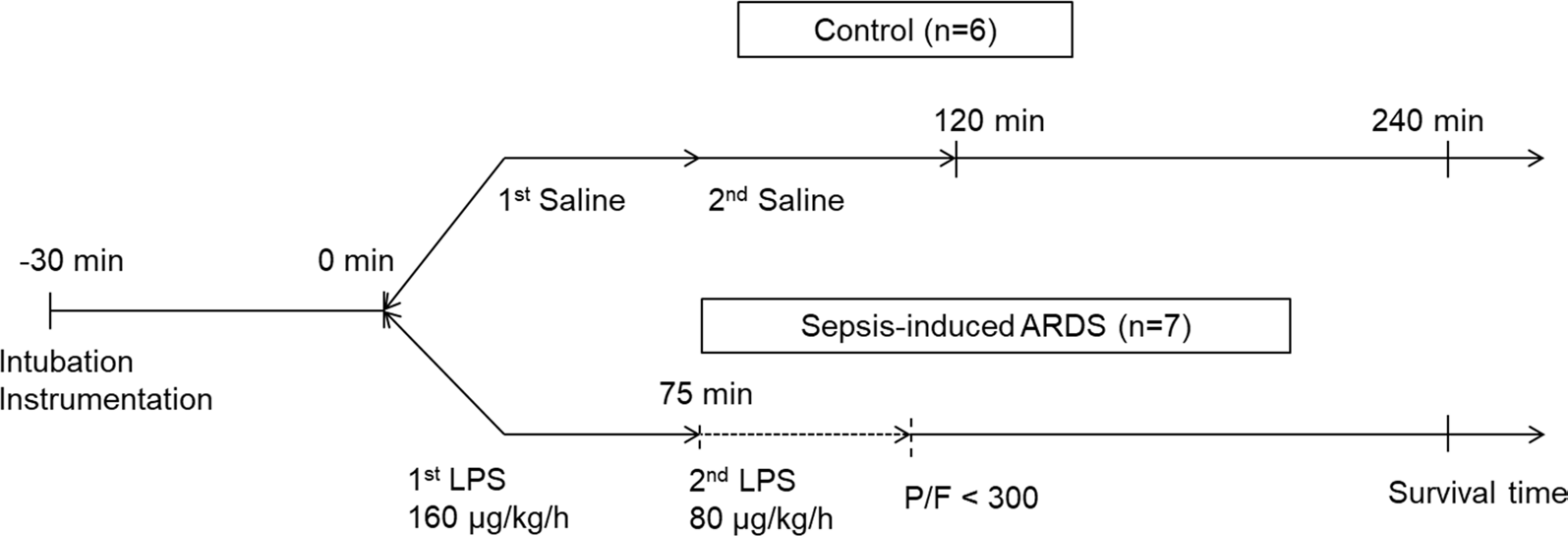
Experimental protocol

We developed a novel model of septic shock and ARDS in which the animals were exposed to a “two-hit” LPS injection, thus exhibiting hypodynamic shock, hypoxia, systemic inflammation, and acute lung injury. The animals were mechanically ventilated with zero positive end-expiratory pressure (PEEP) and a fraction of inspired oxygen (F_I_O_2_) of 1.0 throughout the experiment. End-tidal carbon dioxide was maintained at 40 ± 5 mmHg during the experiment, by the adjustment of respiratory rate and tidal volume. Following hemodynamic stabilization for 30 min, septic-ARDS (n = 7) was induced by intravenous infusions of LPS, which was purified from *Escherichia coli* 055:B5 by ion-exchange chromatography (product number L4524, Sigma Aldrich, St Louis, MO, USA), beginning at 160 µg/kg/h and continued for 75 min. Subsequently, a second dose of LPS was infused as a “second hit” at 80 µg/kg/h, which continued until reaching a ratio of arterial partial oxygen pressure to fractional inspired oxygen (PaO_2_/F_I_O_2_ (P/F)) of < 300 mmHg (Fig. 1). The control group (n = 6) was equally subjected to two infusions of a similar volume of saline using the same infusion rate and timing as for the LPS infusion. TPTD measurements and arterial blood sampling were performed every 30 min. The survival rate was recorded for 4 h after the second dose of LPS. Subsequently, lung status was categorized as normal (P/F ≥ 400) or injured at different degrees: pre-ARDS (300 ≤ P/F < 400), mild-to-moderate ARDS (100 ≤ P/F < 300), or severe ARDS (P/F < 100).

### Hemodynamic measurements

In all animals, we measured the TPTD parameters using the PiCCO monitoring system [22]. TPTD was precisely conducted as previously described [22]. Briefly, (1) we used 10 mL of 0 °C physiological saline 0.9% as an indicator, (2) use of average of three times values, (3) the indicator was injected by the same operator (YE) throughout the experiment, (4) the indicator was injected for 2–3 s, (5) ΔT in TPTD (the change in blood temperature after indicator injection) was recorded; optimal = ΔT > 0.3, good = ΔT > 0.2, and bad = ΔT < 0.2, to verify the reliability of the measurement method. We recorded the stroke volume, cardiac output, mean arterial pressure (MAP), heart rate, systemic vascular resistance (SVR), global end-diastolic volume index (GEDI), stroke volume variation (SVV), pulse pressure variation (PPV), global ejection fraction, PVPI, and ELWI. ELWI was calculated from the absolute volume of EVLW divided by the actual body weight, whereas GEDV and CO are indexed using body surface area (BSA) calculated from body height and weight (GEDI and Cardiac Index, respectively) [23]. The body surface area (BSA) of animal was calculated by the following equation: BSA (m^2^) = 0.0734 x body weight^0.656^ [24]. Simultaneously, we monitored a perfusion index for the sepsis-induced ARDS model (Masimo SET Radical-7TM, Masimo, Irvine, CA, USA).

### Blood gas analysis

Arterial blood sample was collected from the femoral artery at each measurement point to monitor PaO_2_, arterial carbon dioxide, and lactate levels (ABL-90 FLEX; Radiometer, Copenhagen, Denmark). We used the central venous blood gas for measuring the central venous oxygen saturation (ScvO_2_).

### Measuring serum inflammatory cytokines and high mobility group box 1

Whole blood (0.5 mL) was collected from the arterial line into Microtainer^®^ tubes (Becton Dickinson, Franklin Lakes, NJ, USA) with sera separator gel, and centrifuged at 3,000 *g* for 15 min to obtain the serum. The serum samples were stored at −80 ˚C until use. Simultaneously, we measured the serum concentrations of interleukin (IL)-1β, IL-4, IL-6, IL-8, IL-10, IL-12p40, interferon (IFN)-α, IFN-γ, and tumor necrosis factor (TNF)-α using the Bio-Plex Suspension Array System (Bio-Rad, Hercules, CA, USA) with the Porcine Cytokine & Chemokine 9-Plex ProcartaPlex^™^ Panel 1 (Invitrogen, Waltham, MA, USA), according to the manufacturer’s instruction. Furthermore, we measured serum high mobility group box 1 (HMGB1) concentrations by the HMGB1 ELISA Kit II (Shino-Test, Tokyo, Japan) according to the manufacturer’s instruction.

### Computed tomography imaging

To confirm the development of ARDS, we performed computed tomography (CT) imaging in randomly selected two animals which were subjected to the LPS injections. Prior to the CT imaging, we specified the following criteria for ARDS: (1) impaired oxygenation (P/F < 300); (2) the severity of diffuse bilateral opacities on chest CT; and (3) DAD in histopathology [25, 26]. We acquired CT images of the lungs using a 16-slice CT scanner (BrightSpeed Elite SD, GE Healthcare, Chicago, IL, USA), with a scanning range from the apex to diaphragm. Images were acquired with following protocol: 120-kVp, 300-mAs, collimation width 16 × 1.25 mm, pitch factor 1.375, field of view 300 × 300 mm, rack rotation time 1.0 s/r, slice width 1.25 mm, and no intervals, reconstructed with a chest. All images were observed with optimized settings for lung evaluation (window width, 1500 HU; window level, −600HU) and analyzed by a veterinary radiologist. We assumed − 100 Uh above as nonaerated lung tissue and the region below − 100 HU as aerated lung tissue in three-dimension (3D) CT analysis [27].

### Histopathological analysis

For all animals, the veterinary pathologist examined the post-mortem lung tissue for identifying the key features of DAD. Tissues were harvested at 420 min after starting injection of saline or LPS. Lung tissue slices (3 μm thickness) were obtained and stained with hematoxylin and eosin (HE) for histological evaluation. In addition, immunohistochemical staining using anti-pan cytokeratin antibody (AE1/AE3) was performed for visualization of alveolar epithelial cells.

### Statistical analysis

Values are expressed as mean ± standard deviation. We performed a two-sample t-test or Mann-Whitney U test to compare two independent groups, as appropriate. The one-way analysis of variance (ANOVA) followed by Sidak’s correction for post-hoc comparisons was performed for post-hoc comparisons. We examined the TPTD and laboratory data using a mixed-effects model for repeated-measures analyses, followed by ANOVA with Sidak’s correction for post hoc comparisons. Spearman’s correlation coefficients (r) were calculated to evaluate the correlation between each parameter. To examine the accuracy of the ELWI and PVPI for detecting acute lung injury, we performed receiver operating characteristic (ROC) curve analyses. We compared the area under the ROC curve (AUCs) between two pairs of potential predictors by a non-parametric test [28]. A two-sided *P* < 0.05 was considered statistically significant. All statistical analyses were performed using the GraphPad Prism, version 8.3.0 (GraphPad Software, San Diego, CA).

## RESULTS

### Changes in TPTD parameters, lung physiology, and serum inflammatory mediators in sepsis-induced ARDS

**Fig. 2:**
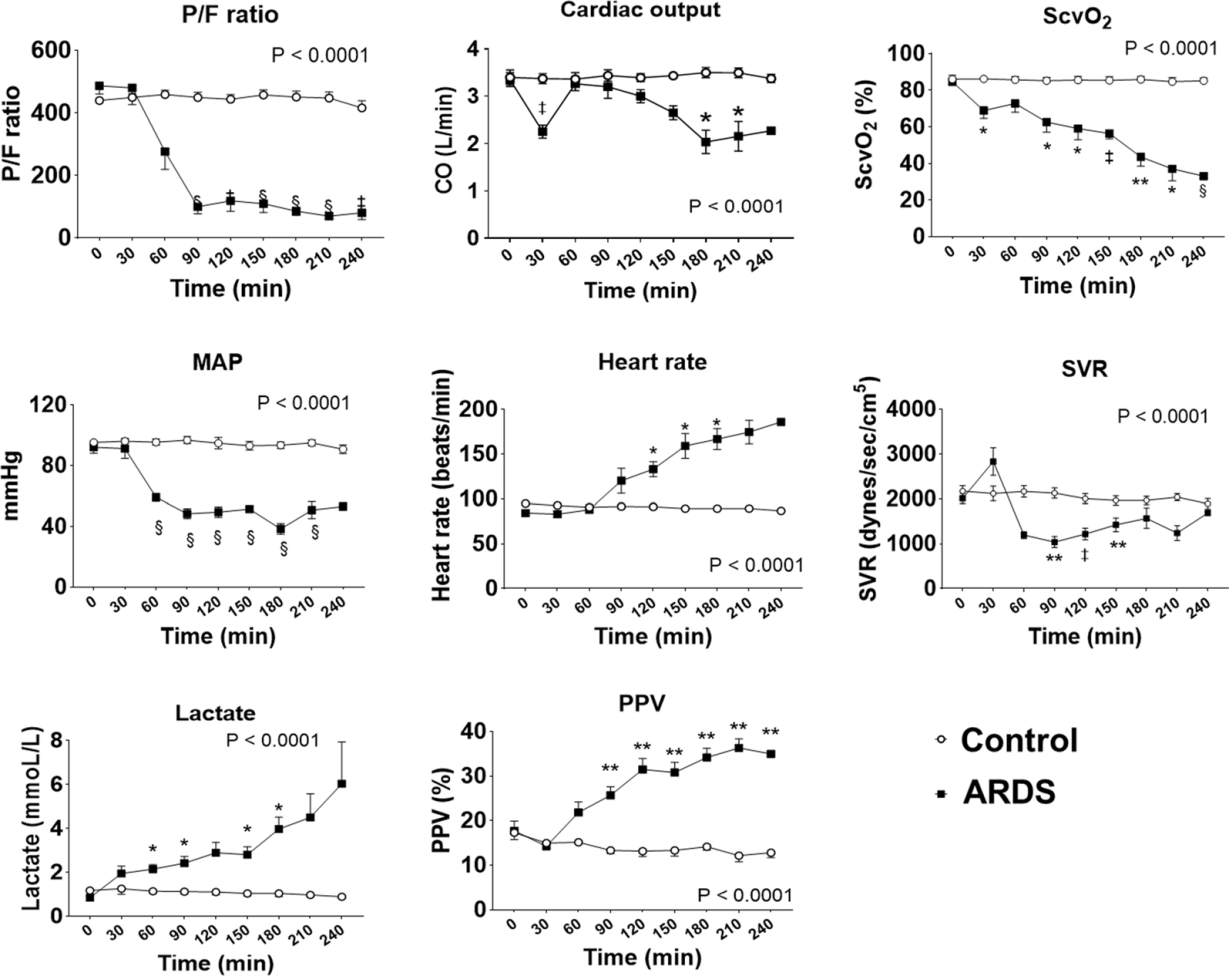
Changes in the partial pressure of oxygen/ fraction of inspired oxygen (P/F), cardiac output, central venous oxygen saturation (ScvO_2_), mean arterial pressure (MAP), heart rate, systemic vascular resistance (SVR), arterial lactate, and pulse pressure variation (PPV) between the control and ARDS groups. n = 6 for the control group; n = 7 for the ARDS group. Changes overtime in all hemodynamic parameters in the ARDS group significantly differed from those in the control group (mixed-effect model, *P* < 0.0001 for all). *P < 0.05, **P < 0.01, ^‡^*P* < 0.001, and ^§^*P* < 0.0001 between each group at the same time points

**Fig. 3:**
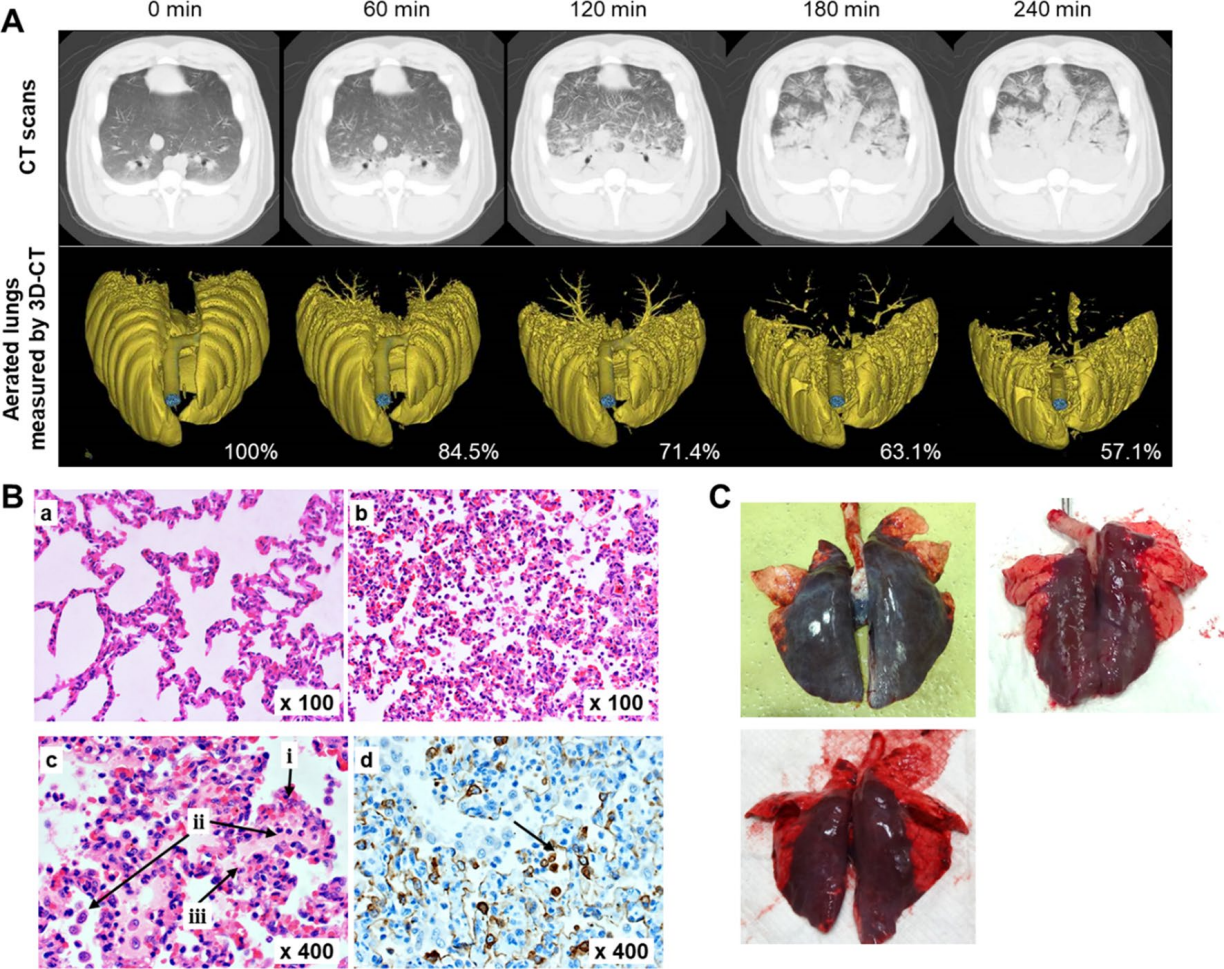
**A** Representative chest computed tomography (CT) images and the aerated lungs images measured by three-dimension CT in the ARDS group at baseline and every 60 min during the experiment. **B** Representative histopathology across experimental groups. a), b), and c): hematoxylin and eosin staining; (a) Normal airway and alveoli in the control group. (b) Dilation of alveolar capillaries with congestion, (c) Infiltration of inflammatory cells mainly composed of neutrophils and macrophages into the alveoli, leakage of eosinophilic proteinaceous materials and precipitation of fibrin in the alveolar space in the ARDS group. The arrows indicate: (i) the dilation and congestion of alveolar capillaries, (ii) infiltration of neutrophils and macrophages, (iii) leakage of eosinophilic proteinaceous materials and precipitation of fibrin. d) Immunohistochemistry with anti-pan-cytokeratin antibody and counterstained with hematoxylin showing shedding of alveolar epithelial cells in the alveolus. The arrow indicates a shedding alveolar epithelial cell. **C** Representative gross pulmonary findings at the end of the experiments demonstrate severe pulmonary hemorrhage and edema

We assessed the hemodynamics changes and lung physiology in the sepsis-induced ARDS model. The 4-h survival rate was 100% in the control group, while 28.6% in ARDS groups (log-rank P = 0.012). Changes overtime in all hemodynamic parameters in the ARDS group significantly differed from those in the control group (mixed-effect model, P < 0.0001 for all) (Fig. 2 and Additional file 1: Figure S1). Animals in the acute lung injury group reached markedly lower P/F, MAP, ScvO_2_, SVR, and higher PVPI, higher heart rate, lactate, PPV within 90 min than those in the control group (Fig. 2). Regarding CT imaging, animals in the ARDS group presented an extent of consolidation in bilateral gravitationally dependent gradient that expands over time, with diffuse ground-glass opacification (Fig. 3A). In the 3D-CT analysis, aerated lung volume decreased from 100% of the baseline volume to 84.5%, 71.4%, 63.1%, and 57.1% at 60, 120, 180, and 240 min, respectively (Fig. 3A). For lung histological and immunohistochemical findings, dilation of alveolar capillaries with congestion, infiltration of inflammatory cells mainly composed of neutrophil and macrophage infiltrates in alveoli, leakage of eosinophilic proteinaceous materials and precipitation of fibrin in the alveolar space, as well as shedding of alveolar epithelial cells positive for anti-pan cytokeratin antibody in the alveoli were conspicuous in the ARDS groups, but not in the control group (Fig. 3B). Figure 3C represents gross pulmonary findings at the end of experiment in animals subjected to LPS injection. Gross lung sections in the ARDS group show severe pulmonary hemorrhage and edema. These findings indicate that our model recapitulated the physiologic, radiographic, and histopathologic features of human ARDS.

### PVPI and ELWI assessments in the ARDS during septic shock

**Fig. 4:**
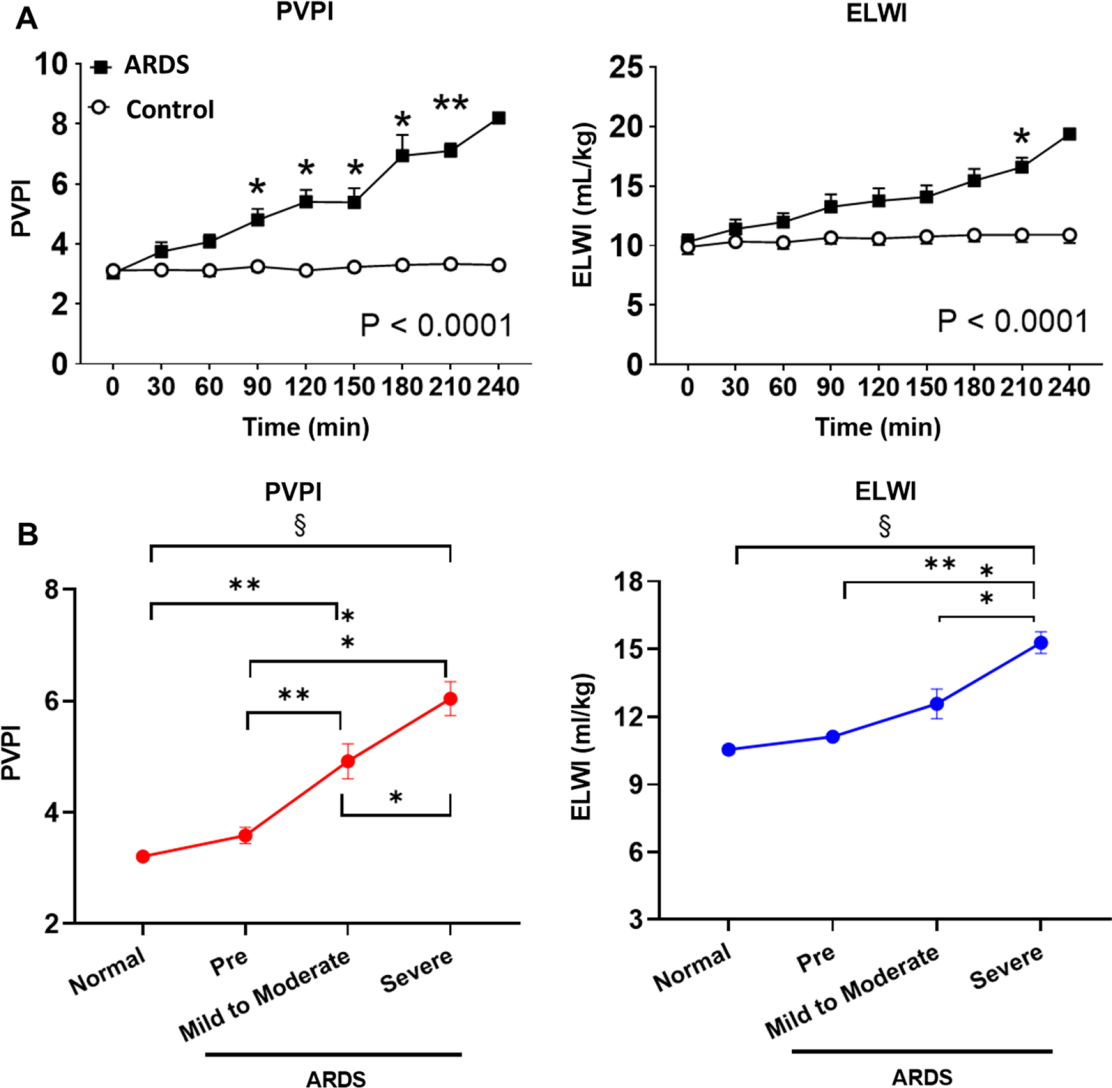
**A** Changes in the pulmonary vascular permeability index (PVPI) and extravascular lung water index (ELWI) (mixed-effect model, P < 0.0001 for both). **B** Changes in the PVPI and ELWI among the statuses. There are positive linear trends between the severity of ARDS and the PVPI or ELWI (analysis of variance for trend test, P < 0.0001 for both). *P < 0.05, **P < 0.01, and ^§^*P* < 0.0001 between each status. n = 90 for the normal, n = 8 for pre, n = 11 for mild to moderate, and n = 21 for severe ARDS

PVPI and ELWI in the ARDS group increased over time (Fig. 4A). We identified a positive linear trend between the PVPI and the severity of ARDS (ANOVA for trend, P < 0.0001), with the highest (6.2 ± 0.3) and lowest (3.2 ± 0.0) PVPI values in the severe ARDS and normal statuses, respectively (Fig. 4B). Moreover, the ELWI increased significantly with the severity of ARDS (P < 0.0001) (Fig. 4B).

**Fig. 5:**
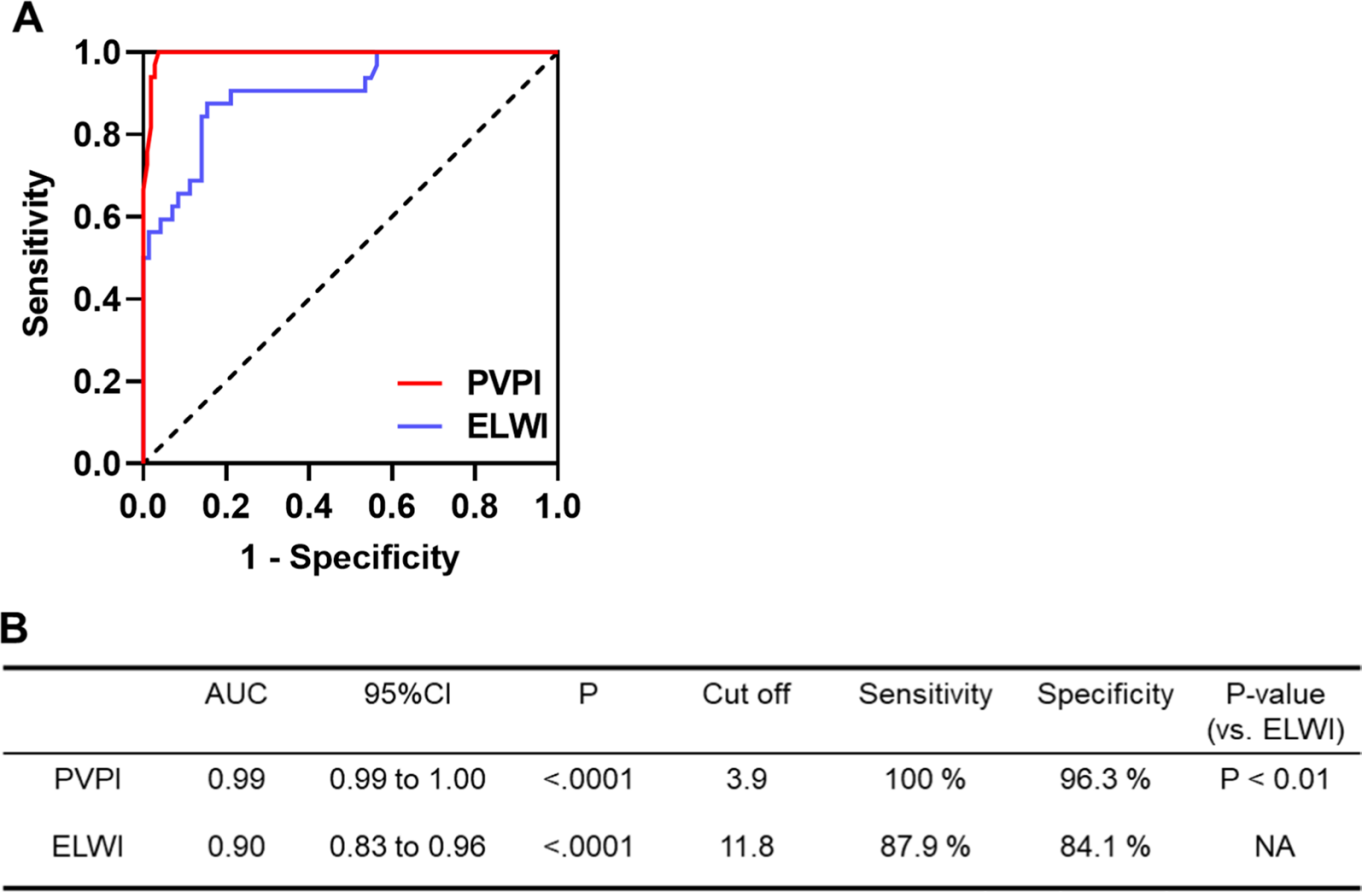
**A** Receiver operating characteristic curves for pulmonary vascular permeability index (PVPI) and extravascular lung water index (ELWI) to diagnose the ARDS (including mild to moderate and severe lung injury) **B** Characteristics of prognostic indexes for PVPI and ELWI. * ROC* receiver operating characteristic curve, *AUC* area under the ROC curve

**Fig. 6:**
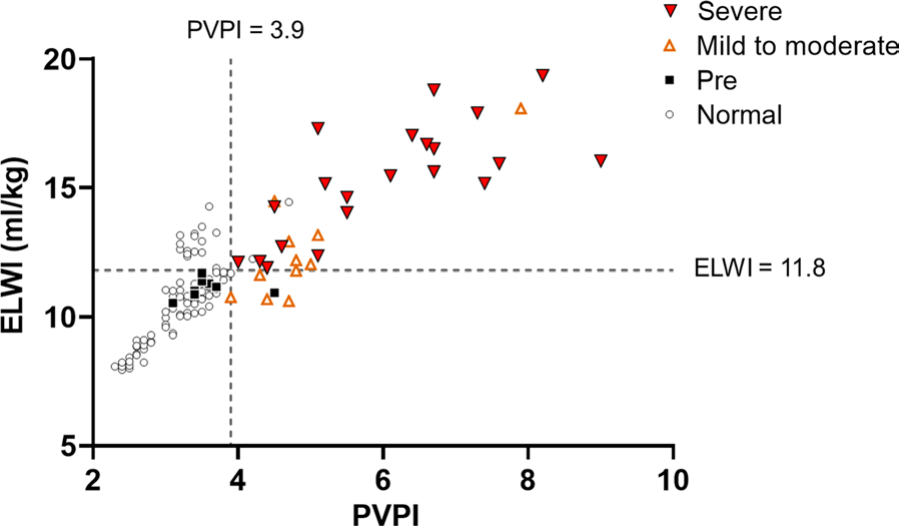
Plots of pulmonary vascular permeability index (PVPI) and extravascular lung water index (ELWI) according to the ARDS severity status in all animals. The four different markers represent normal lung status (P/F ≥ 400, open circles), pre (300 ≤ P/F < 400, closed squares), mild-to-moderate (100 ≤ P/F < 300, closed squares) and severe ARDS (P/F < 100, open triangle). The optimal PVPI and ELWI cut-offs for predicting the ARDS (including mild to moderate and severe lung injury status) are 3.9 and 11.8 (n = 90 for normal, n = 8 for mild, n = 11 for moderate, and n = 21 for severe ARDS).

We plotted ROC curves to determine the values of the PVPI and ELWI for predicting the ARDS severity (Fig. 5A). AUCs of the PVPI and ELWI were 0.99 (95% CI 0.98–1.00; P < 0.0001) and 0.93 (0.88–0.98; P < 0.0001), respectively, for predicting the development of ARDS. The PVPI cut-off of 3.9 provided the optimal sensitivity and specificity (sensitivity = 100%, specificity = 92.5%). We also identified the ELWI cut-off of 11.8 mL/kg as the optimal value (sensitivity = 87.5%, specificity = 89.2%). The AUC for PVPI was significantly superior to that for ELWI, with respect to ARDS diagnosis (P < 0.01, Fig. 5C). In animals showing ARDS (P/F < 300), AUCs of the PVPI and ELWI were 0.76 (0.58–0.94; P = 0.01; optimal cut-off, 5.1) and 0.83 (0.67–1.00; P < 0.001; optimal cut-off, 13.6), respectively, for distinguishing between mild to moderate (100 ≤ P/F < 300) and severe ARDS (P/F < 100). Figure 6 presents the plots for all animals according to the severity of ARDS

**Fig. 7:**
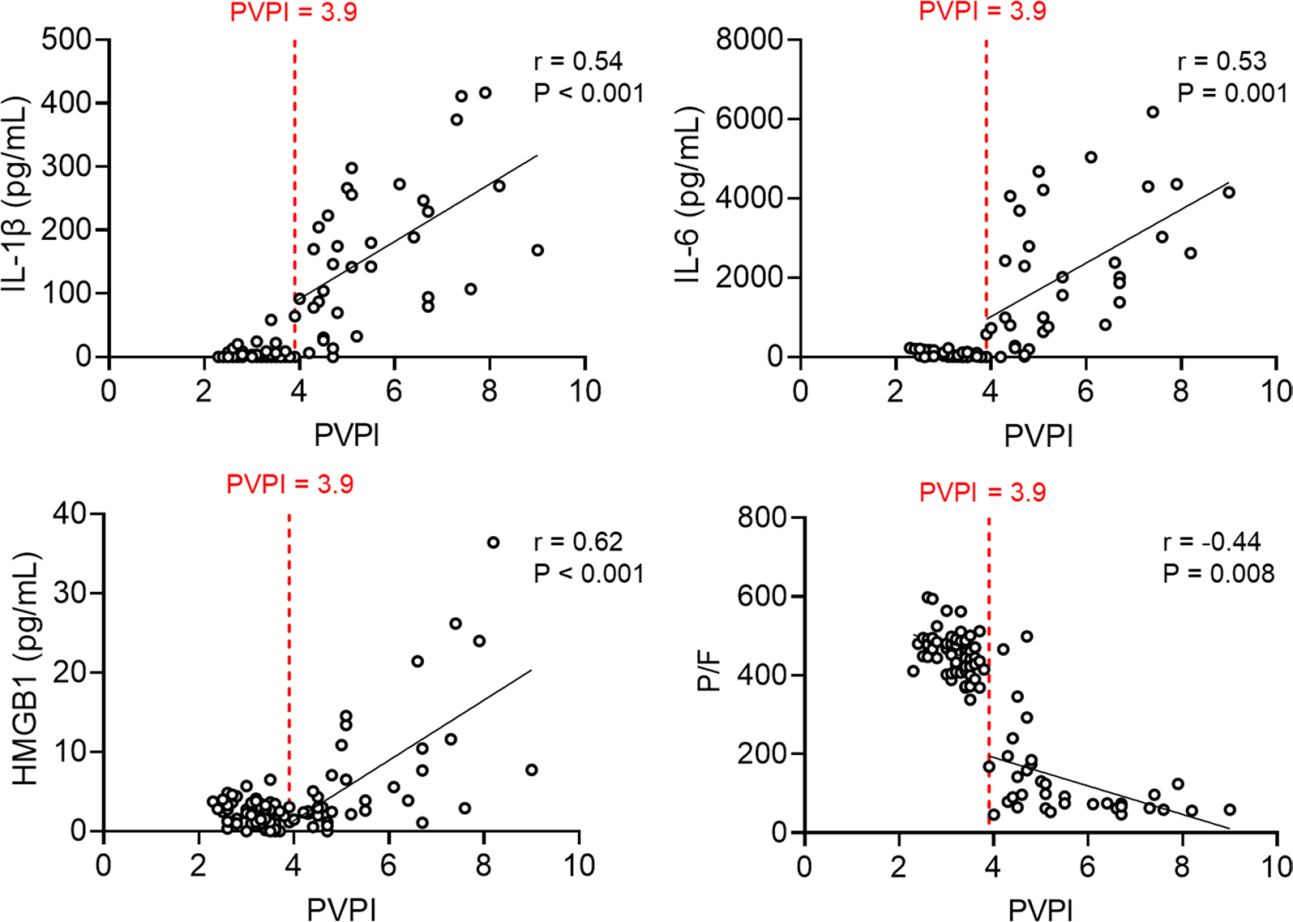
Significant correlations between pulmonary vascular permeability index (PVPI) and serum inflammatory markers or P/F ratio in a subgroup of animals with PVPI > 3.9. IL, interleukin; HMGB1, high mobility group box 1

**Fig. 8:**
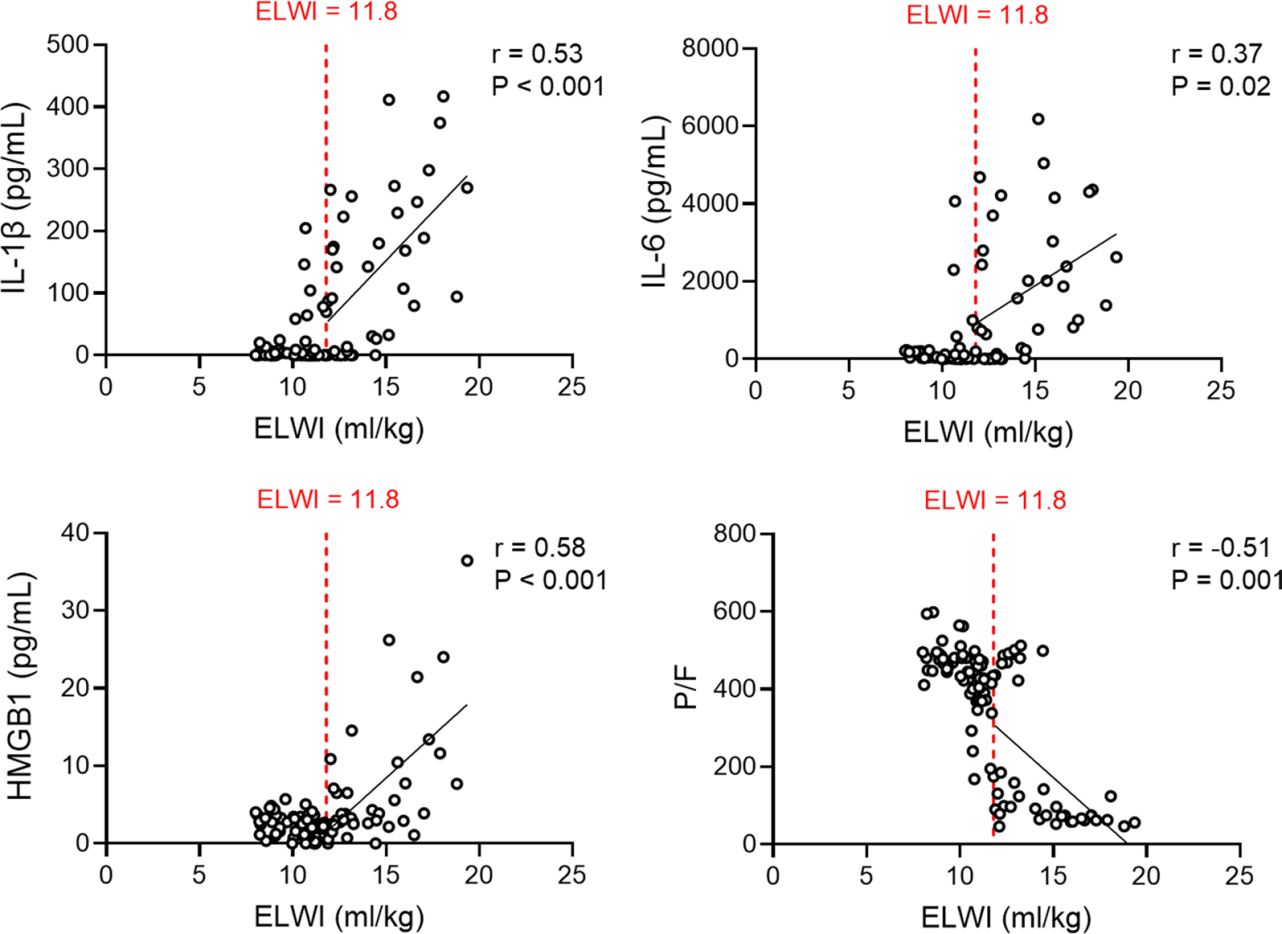
Significant correlations between extravascular lung water index (ELWI) and serum inflammatory markers or P/F ratio in a subgroup of animals with ELWI > 11.8. IL, interleukin; HMGB1, high mobility group box 1

Serial changes in inflammatory cytokine, HMGB1, and hematocrit levels and platelet counts during the experiment are presented in Additional file 1: Figure S2. For all markers, there were significant differences in serial changes over time between the control and ARDS groups (mixed-effect model, P < 0.0001 for all, except IFN-α [P = 0.043]) ( Additional file 1: Figure S2. To further investigate the role of PVPI in the development of ARDS, we analyzed its association with serum inflammatory mediators and P/F. Animals with PVPI > 3.9 displayed significant positive correlations between the PVPI and IL-1β, IL-6, and HMGB1 (r = 0.54, P < 0.001; r = 0.53, P = 0.001; r = 0.62, P < 0.001, respectively) and negative correlations between the PVPI and P/F (r = −0.44, P = 0.008) (Fig. 7). Animals with ELWI > 11.8 displayed significant positive correlations between the ELWI and IL-1β, IL-6, and HMGB1 (r = 0.53, P < 0.001; r = 0.37, P = 0.02; r = 0.58, P < 0.001, respectively) and negative correlations between the ELWI and P/F (r = −0.51, P = 0.001) (Fig. 8). Additional file 1: Figure S3 and S4 depicts the relationships of the PVPI and ELWI with other inflammatory makers.

## Discussion

This study provides clear evidence that both PVPI and ELWI significantly increased with the severity of ARDS related to LPS-induced septic shock. PVPI has shown to be superior to ELWI when considering their diagnostic properties regarding ARDS. In this pig model, the PVPI cut-off of 3.9 provided optimal sensitivity and specificity for distinguishing ARDS from a normal lung status (AUC, 0.99; 95% CI 0.98–1.00; P < 0.0001; sensitivity = 100%, specificity = 92.5%). Furthermore, the PVPI was significantly correlated with increased serum inflammatory mediators and worsened oxygenation in animals developing ARDS. These novel findings are essential for the management of patients with septic ARDS and support the hypothesis that the PVPI can be a reliable indicator of ARDS severity during septic shock.

The most important findings in the current study are that PVPI is well associated with multiple parameters that reflect clinicopathological changes in animals with ARDS. To the best of our knowledge, human or animal research has been very limited so far in verifying the validity of TPTD measurements to assess extravascular lung water and pulmonary vascular permeability during septic shock-induced ARDS. Importantly, an early diagnosis and assessment of ARDS severity will largely improve the outcome in patients with ARDS [29, 30]. Our findings are therefore crucial because no direct or highly reproducible approach assessing pulmonary vascular permeability exists in the current therapeutic strategy for patients with ARDS.

Currently, the “Berlin definition” does not include the ELWI or PVPI [25, 31] owing to concerns over arbitrary cut-off values, methodological concerns, and limited availability worldwide [32]. However, several experts have proposed objective diagnoses for ARDS using ELWI and PVPI [11, 12] and the inclusion of both parameters in a future definition [33, 34]. We observed a significant increase in the PVPI along with worsened oxygenation during septic ARDS, consistent with previous reports [14, 18, 35]. Based on the results from large clinical studies on TPTD techniques in ARDS [14, 18, 36, 37], critical care experts have proposed that a PVPI < 2 may represent normal pulmonary permeability, whereas that > 3 indicates leaky lungs [12]. By contrast, the cut-off value for ARDS obtained in the current study was higher than that in previous human studies. This discrepancy could be explained by the difference in species. Further studies are required to understand the noticeable differences between human ARDS and pig model of acute lung injury in terms of PVPI value in several conditions.

Based on evidence from pathological and clinical studies [15, 37–39], a normal ELWI does not exceed 10 ml/kg and one > 10 mL/kg is a reasonable threshold for pulmonary edema. An increase in ELWI > 15 mL/kg identified patients with DAD with 99% certainty [15]; therefore, researchers have proposed an ELWI > 15 mL/kg to indicate severe pulmonary edema [12]. Our current study demonstrated similar results that the ELWI was 10.5 ± 0.2 mL/kg in animals with normal lung, compared with 15.3 ± 0.5 mL/kg in those with severe ARDS, thus highlighting the accuracy of the TPTD-based ELWI measurement.

Interestingly, the absence of adequate preclinical animal models to study the syndrome is a major barrier to improved ARDS diagnosis and management [40]. No single animal model satisfactorily reproduces all histopathological elements of ARDS [41]. In the current study, we used the novel septic-ARDS model and confirmed that the LPS “two-hit” infusions could reproductively induce DAD, systemic inflammation, distributive shock, and clinically relevant physiological characteristic of sepsis. LPS or endotoxin induced-ARDS pig model is relatively reproducible [42] and has clinical relevance in terms of the etiology [43], direct damages to and subsequent apoptosis of the endothelial cells by endotoxin [42, 44], and the activation of a systemic inflammatory response [45]. Furthermore, a recent international consensus by a multidisciplinary working group from the National Heart, Lung, and Blood Institute has recommended the use of large animal models of ARDS rather rodent models, owing to their genetic and physiological similarities with human and greater potential for clinical translation [40]. Recently, Tiba et al. introduced a novel swine model of ARDS in which combination of indirect (injection of *Escherichia coli* into the kidney) and direct lung injuries (volutrauma, hyperoxia, and bronchoscope-delivered gastric particles) faithfully recapitulated the physiologic, radiographic, and histopathologic features of human ARDS [26]. By contrast, we used only LPS infusions to easily reproduce the clinically relevant features of human ARDS. It likely fills a crucial gap in the translational study of clinical ARDS.

Our study had some limitations that should be addressed in the future. First, the sample size was small and could have resulted in a type 1 error, thus warranting further studies to confirm our findings. Second, we only included female animals to minimize heterogeneity. Females are more likely than males to develop ARDS [46]; therefore, future studies should include both males and females to understand the association between acute lung injury and the sex hormones. Third, the current study did neither obtain data regarding long-term changes in ELWI or PVPI nor proposed an explanation for the pathophysiological mechanisms underlying ARDS and changes in associated parameters. For example, the association between prone positioning and PVPI values during ARDS was not investigated. Eventually, the animals in our model were ventilated without PEEP to easily induce atelectrauma, which could have affected our results. ARDS severity was determined by P/F; however, PEEP could have affected the P/F [47]. Finally, it is considered that it is still early to apply this cut-off value to human in clinical. Similar to previously study, in our experiments, the baseline value of PVPI in pigs may be higher than in human [48].

In conclusion, the current study demonstrated that PVPI is a valid indicator to quantify the LPS-induced septic ARDS severity. The usefulness of PVPI value to diagnose and manage ARDS during septic shock should be acknowledged by clinicians. This is a novel and important finding that supports additional translational research for the management of septic ARDS. Further evaluations are required in clinical settings to continue assessing its usefulness and ability for improving the outcomes in patients with septic ARDS.

## Supplementary Information

**Additional file 1:** **Figure S1.** Changes in hemodynamic parameters (mixed-effect model, P < 0.0001 for all). n=6 for the control group, n=7 for ARDS group. * P < 0.05, ** P < 0.01, ‡ P < 0.001, and § P < 0.0001 between each group. SV, stroke volume; GEF, global ejection fraction; GEDI, global end-diastolic volume index; and SVV, stroke volume variation. **Figure S2.** Changes in inflammatory markers, hematocrit, and platelet counts during the experiment (mixed-effect model, P < 0.0001 for all). n=6 for the control group, n=7 for ARDS group. * P < 0.05, ** P < 0.01, ‡ P < 0.001, and § P < 0.0001 between each group. **Figure S3.** Correlations between the pulmonary vascular permeability index (PVPI) and experimental parameters in a subgroup of animals with PVPI >3.9. **Figure S4.** Correlations between extravascular lung water index (ELWI) and experimental parameters in a subgroup of animals with ELWI > 11.8.

## Data Availability

The datasets used and/or analyzed during the current study are available from the corresponding author on reasonable request.
