# Supplementary material for: Diagnostic value of transpulmonary thermodilution measurements for acute respiratory distress syndrome in a pig model of septic shock
Source: J Transl Med. 2022 Dec 23;20:617. doi: 10.1186/s12967-022-03793-x (PMC9789654; doi:10.1186/s12967-022-03793-x)
Supplement: Supplementary file 1 — Additional file 1: Figure S1. Changes in hemodynamic parameters (mixed-effect model, P < 0.0001 for all). n=6 for the control group, n=7 for ARDS group. * P < 0.05, ** P < 0.01, ‡ P < 0.001, and § P < 0.0001 between each group. SV, stroke volume; GEF, global ejection fraction; GEDI, global end-diastolic volume index; and SVV, stroke volume variation. Figure S2. Changes in inflammatory markers, hematocrit, and platelet counts during the experiment (mixed-effect model, P < 0.0001 for all). n=6 for the control group, n=7 for ARDS group. * P < 0.05, ** P < 0.01, ‡ P < 0.001, and § P < 0.0001 between each group. Figure S3. Correlations between the pulmonary vascular permeability index (PVPI) and experimental parameters in a subgroup of animals with PVPI >3.9. Figure S4. Correlations between extravascular lung water index (ELWI) and experimental parameters in a subgroup of animals with ELWI > 11.8. [file 12967_2022_3793_MOESM1_ESM.pdf]

Diagnostic value of transpulmonary thermodilution measurements for acute respiratory distress syndrome in a pig model

Yusuke Endo, Taku Miyasho, Kanako Endo, Yoshio Kawamura, Kenjiro Miyoshi, Ryosuke Takegawa, Takashi Tagami, Lance B. Becker, Kei Hayashida

Supplementary materials

Supplemental figure legends

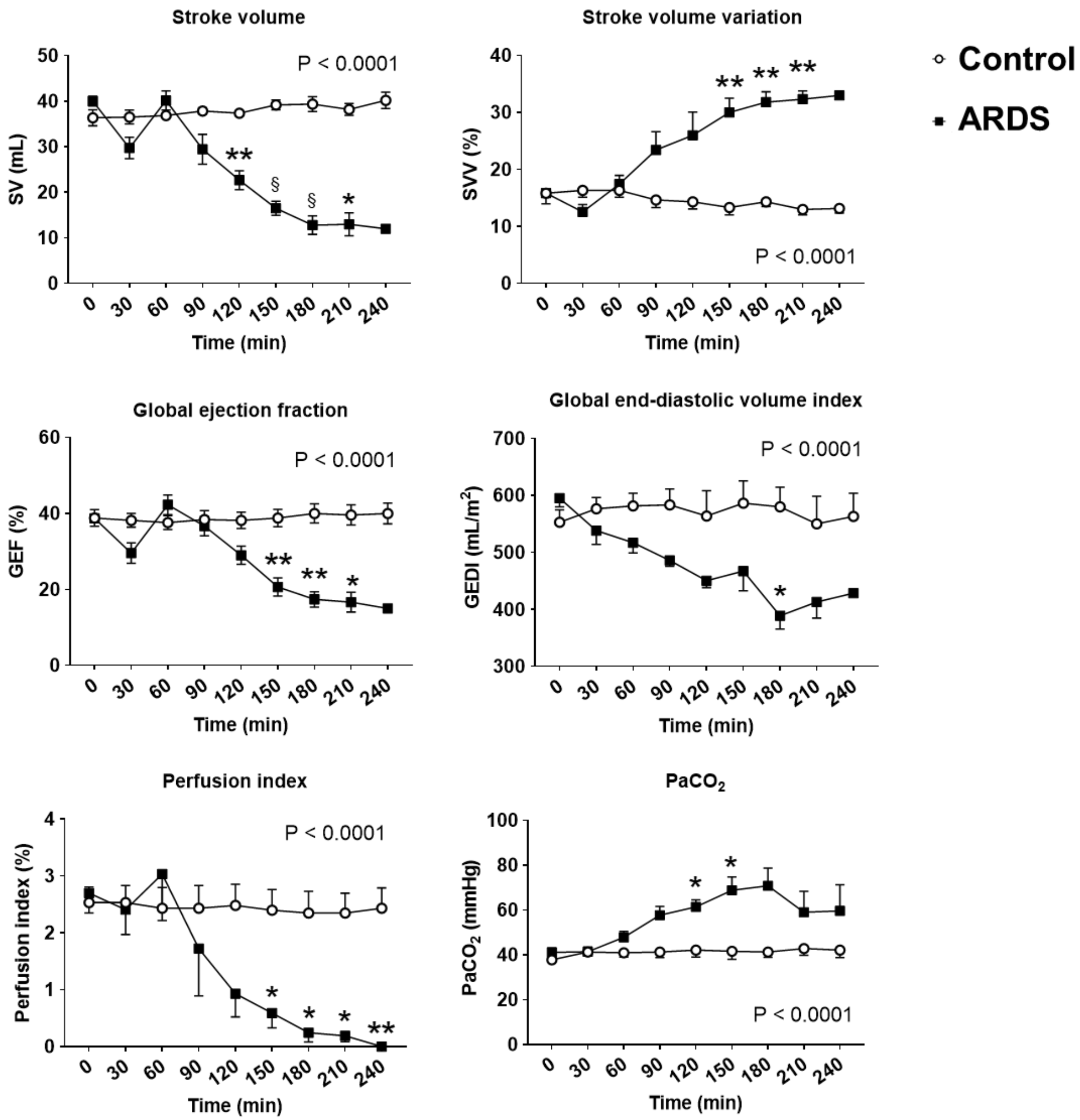

Supplementary Figure 1. Changes in hemodynamic parameters (mixed-effect model, P < 0.0001 for all). n=6 for the control group, n=7 for ARDS group. * $P < 0.05$, ** $P < 0.01$, ‡ $P < 0.001$, and § $P < 0.0001$ between each group. SV, stroke volume; GEF, global ejection fraction; GEDI, global end-diastolic volume index; and SVV, stroke volume variation.

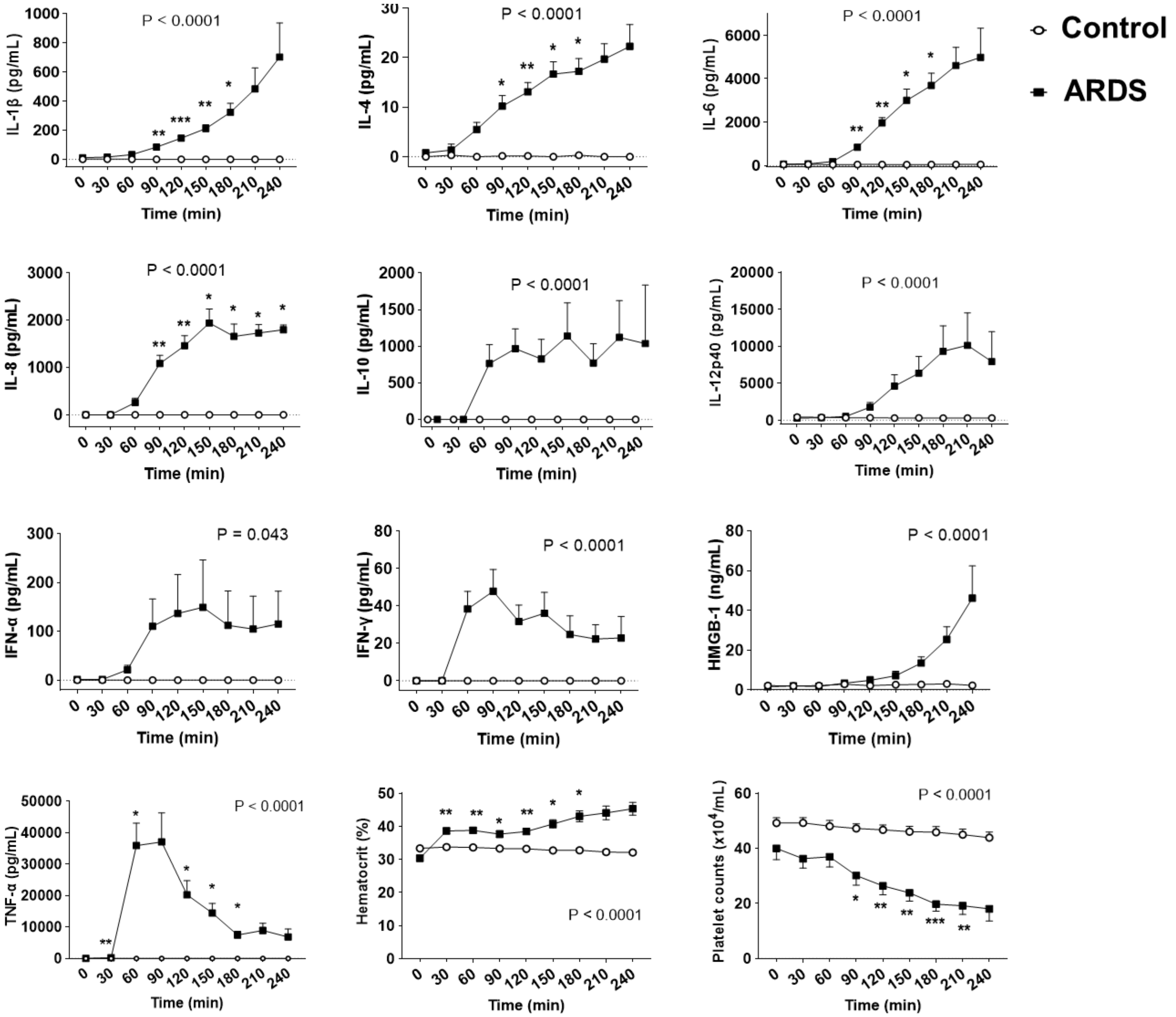

Supplementary Figure 2. Changes in inflammatory markers, hematocrit, and platelet counts during the experiment (mixed-effect model, $P < 0.0001$ for all). n=6 for the control group, n=7 for ARDS group. * $P < 0.05$, ** $P < 0.01$, ‡ $P < 0.001$, and § $P < 0.0001$ between each group.

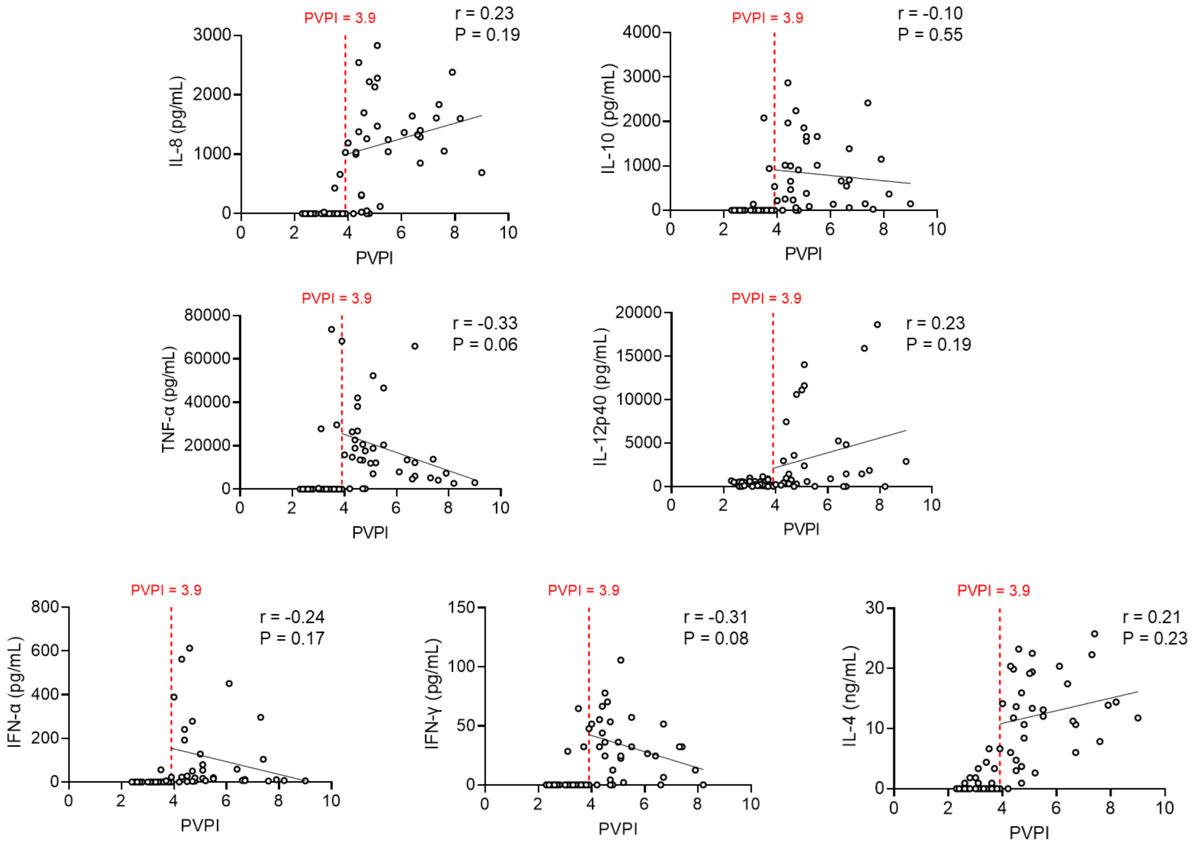

Supplementary Figure 3. Correlations between the pulmonary vascular permeability index (PVPI) and experimental parameters in a subgroup of animals with PVPI >3.9.

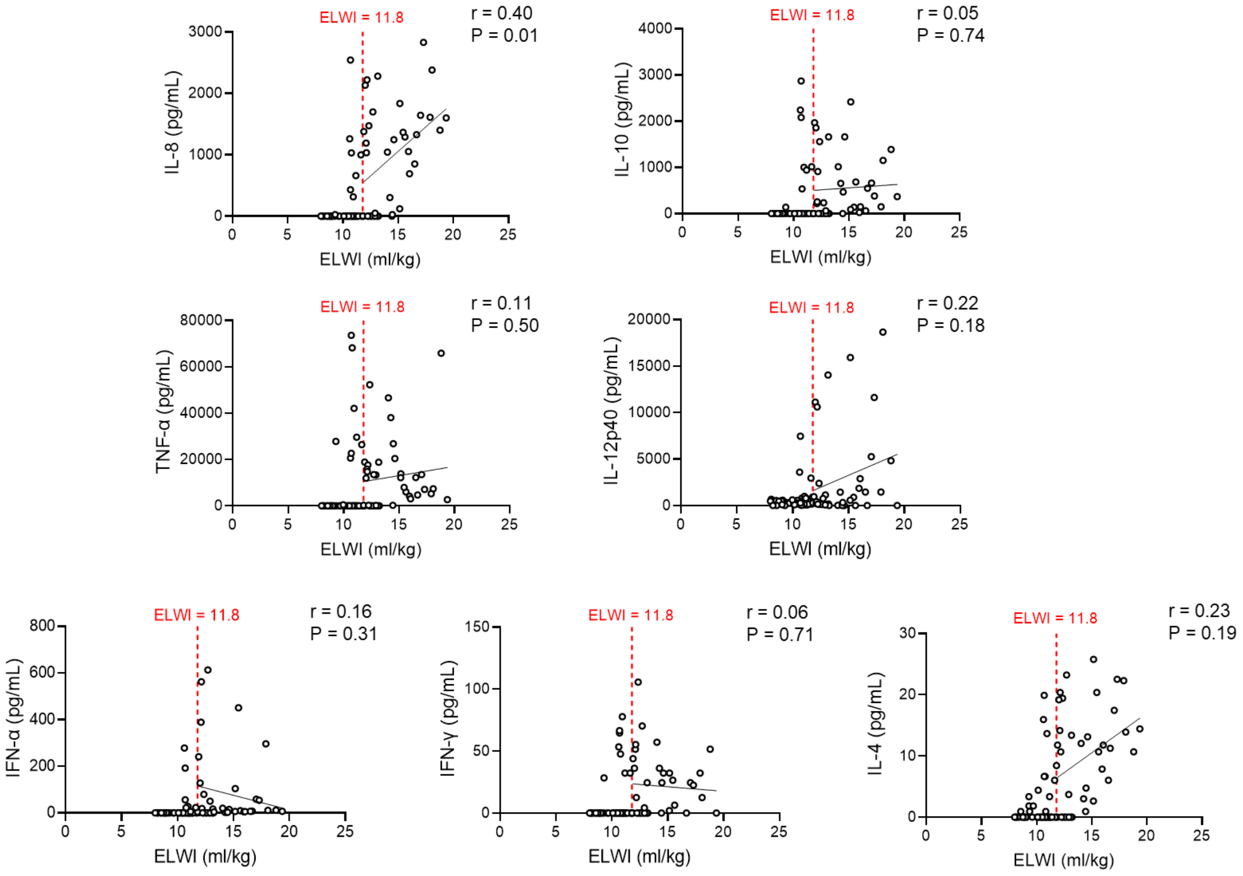

Supplementary Figure 4. Correlations between extravascular lung water index (ELWI) and experimental parameters in a subgroup of animals with ELWI > 11.8.
